# Hyaluronic Acid-Coated Glabridin Nanoemulsions via a Low-Energy Method: Molecular Dynamics Analysis and Enhanced Macrophage Uptake

**DOI:** 10.3390/ijms27104207

**Published:** 2026-05-09

**Authors:** Yotsanan Weerapol, Suwisit Manmuan, Poomipat Tamdee, Jitnapa Sirirak, Tiraniti Chuenbarn, Sukannika Tubtimsri

**Affiliations:** 1 Faculty of Pharmaceutical Sciences, Burapha University, Chonburi 20131, Thailand; yotsanan@go.buu.ac.th (Y.W.); suwisit@go.buu.ac.th (S.M.); tiraniti.ch@go.buu.ac.th (T.C.); 2 Department of Chemistry, Faculty of Sciences, Silpakorn University, Nakhon Pathom 73000, Thailand; tamdee_p@silpakorn.edu (P.T.); sirirak_j@silpakorn.edu (J.S.)

**Keywords:** molecular dynamics study, low energy method, hyaluronic acid-coated nanoemulsion, macrophage, glabridin

## Abstract

In this study, a hyaluronic acid (HA)-coated glabridin nanoemulsion was developed for enhanced macrophage uptake using the phase inversion temperature (PIT) method. The optimized cationic nanoemulsion consisted of a 10% *w*/*w* 60:40 peppermint oil:virgin coconut oil ratio, 10% *w*/*w* Cremophor RH40, 1% *w*/*w* cetyltrimethylammonium bromide, and 1% *w*/*w* ethanol. It exhibited a small droplet size, narrow size distribution, and positive zeta potential. Molecular dynamics simulations supported the experimental results, showing that systems containing a cosurfactant formed smaller droplets than those without a cosurfactant, particularly when ethanol was used as the cosurfactant. A concentration of 0.15% *w*/*w* HA solution at a 1:10 nanoemulsion–HA ratio yielded favorable characteristics, including a small droplet size (69.98 ± 0.48 nm), narrow size distribution (0.30 ± 0.00), and negative zeta potential (−23.00 ± 2.08 mV). Transmission electron microscopy image confirmed the presence of HA coating at 0.15% *w*/*w*. The incorporation of glabridin stabilized the droplet size (67.63 ± 0.33 nm) and polydispersity index (0.36 ± 0.01) but slightly decreased the absolute zeta potential (−10.83 ± 1.91 mV), whereas the entrapment efficiency was 91.65% ± 1.52% *w*/*w*. The nanoemulsion exhibited good physicochemical stability after storage at 40 °C for 6 months. HA coating enhanced the cellular uptake of the nanoemulsion into macrophage cells. The HA-coated glabridin nanoemulsion significantly inhibited the production of reactive oxygen species and nitric oxide and it also demonstrated low cytotoxicity. These findings indicated the potential of the PIT method to produce HA-coated glabridin nanoemulsion as a receptor-mediated delivery system for treating inflammation.

## 1. Introduction

Inflammation is a biological response that functions as a protective mechanism against harmful stimuli and initiates healing. Macrophages are immune cells derived from monocytes (M0) that play a pivotal role in the inflammatory process. They can differentiate into either a proinflammatory (M1) or anti-inflammatory (M2) state. Of the phenotypes, M1 macrophages exhibit the highest surface concentration of hyaluronic acid (HA; CD44) receptor in response to various stimuli, including pathogens and tumors [1]. After activation, M1 macrophages produce proinflammatory cytokines, reactive nitrogen species, and reactive oxygen species (ROS) that can damage tissues, amplify inflammation, and worsen disease [2,3]. HA receptor-mediated delivery of anti-inflammatory agents into macrophages may effectively reduce excessive inflammatory responses.

Glabridin (GLA) is a natural substance found in the root of *Glycyrrhiza glabra* L. (licorice) at concentrations ranging from 0.08% to 0.35% *w*/*w*. This compound is classified as a prenylated isoflavan and was formally known as 4-[(3R)-8,8-dimethyl3,4-dihydro-2H-pyrano[2,3-f]chromen-3-yl]benzene-1,3-diol [4]. GLA exhibits a diverse spectrum of pharmacological effects, including antimicrobial [5], anti-obesity, immune-modulatory [6], and anti-inflammatory [7]. It demonstrates low solubility in water, which significantly limits its practical applications. This limitation can be effectively addressed by incorporating GLA into oil-in-water nanoemulsions, thereby markedly enhancing its water compatibility.

Oil-in-water nanoemulsions have been widely used for poorly water-soluble substances. However, the lack of specific delivery limits their therapeutic efficacy. Recent studies show that interfacial engineering and nanoscale synergy enhance system performance, highlighting the importance of surface properties in nanoemulsion stability and cellular uptake [8,9]. Surface modification with HA, a biocompatible polysaccharide with affinity for CD44 receptors, can overcome the lack of specific delivery. HA-coated nanoemulsions enable receptor-mediated delivery, resulting in enhanced efficacy of the active substance. Previous reports have revealed the successful preparation of HA-coated nanoemulsion. Kim et al. [10] developed hyaluronan-coated nanoemulsions for the delivery of poorly soluble paclitaxel. The nanoemulsions exhibited high drug-loading capacity, long-term stability, and strong CD44-mediated tumor cell targeting. Tinoco et al. [11] reported that HA-coated nanoemulsions of all-trans retinoic acid demonstrated high encapsulation efficiency, stability, and controlled release. It also enhanced cytotoxicity against CD44-overexpressing breast cancer cells by minimizing toxicity. Additionally, Pleguezuelos-Villa et al. [12] successfully developed topical mangiferin-loaded HA nanoemulsions with anti-inflammatory effects on skin lesions in a mouse model.

The low-energy method is commonly used for nanoemulsion preparation due to its lower energy requirement. The phase inversion temperature (PIT) method utilizes the temperature-dependent solubility of nonionic surfactants and polyoxyethylene derivatives. As the surfactant shifts from water- to oil-soluble, the emulsion inverts from oil-in-water to water-in-oil. Several previous studies have reported the success of this technique for the production of nanoemulsions using nonionic surfactants, especially polyoxyethylene surfactant [13,14]. However, ionic surfactants are generally considered unsuitable for the PIT method, as their solubility characteristics are not markedly affected by temperature variations. Consequently, the application of the PIT method has been largely limited to nonionic systems, and the development of ionic nanoemulsions using this approach remains challenging. In particular, the application of the PIT method in combination with polyoxyethylene surfactants and cationic surfactants, such as cetyltrimethylammonium bromide (CTAB), for the fabrication of cationic nanoemulsions has been rarely investigated.

In this study, HA-coated nanoemulsions were developed for the delivery of GLA to macrophage. GLA was selected as a model natural anti-inflammatory agent. The cationic nanoemulsion prior to HA coating was prepared using Cremophor RH40 and CTAB by PIT method. The effects of Cremophor RH40, CTAB and a cosurfactant on the properties of nanoemulsions were investigated by experimental and molecular dynamics (MD) studies.

## 2. Results

### 2.1. Droplet Size and Zeta Potential

**Figure 1:**
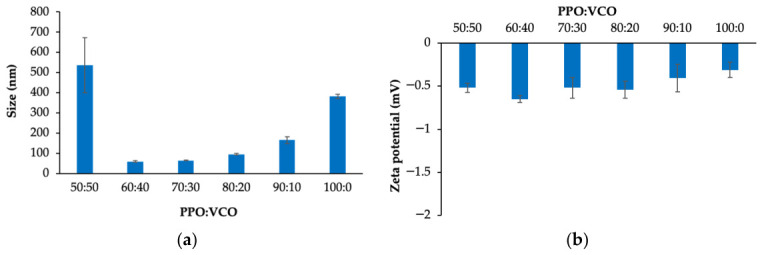
Droplet size (**a**) and zeta potential (**b**) of the nanoemulsions prepared from different peppermint oil:virgin coconut oil (PPO:VCO) ratios.

In this study, peppermint oil (PPO) was selected as the carrier oil due to its anti-inflammatory activity. However, PPO alone was insufficient to produce a stable nanoemulsion. To enhance emulsion stability, a medium-chain or long-chain triglyceride needed to be incorporated. Therefore, virgin coconut oil (VCO), which is primarily composed of medium-chain triglycerides, was selected for this formulation. Various PPO:VCO ratios ranging from 50:50 to 100:0 were evaluated to identify the formulation yielding the smallest droplet size (Figure 1a). PPO:VCO ratios of 60:40, 70:30, and 80:20 produced droplet sizes measuring <100 nm, with the 60:40 ratio yielding the smallest droplet size at 60.83 ± 3.84 nm. The 50:50, 90:10, and 100:0 produced droplet sizes measuring >100 nm.

Besides the droplet size, the zeta potential of all ratios presented near-zero values (Figure 1b). Based on these findings, the 60:40 ratio was selected for further experiments.

### 2.2. Effect of Cetyltrimethylammonium Bromide on Droplet Size, Size Distribution, and Zeta Potential

**Figure 2:**
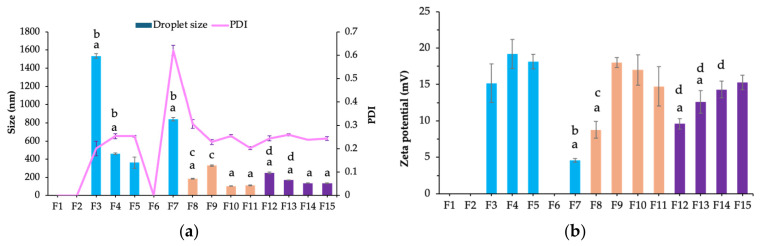
Droplet size, size distribution (polydispersity index [PDI]) (**a**) and zeta potential (**b**) of the nanoemulsions prepared with different compositions. Blue, orange, and purple bars represent the formulations without cosurfactant, with ethanol, and with polyethylene glycol 400, respectively. ^a^ significant difference from F5 (F1 to F15). ^b^ significant difference from F5 (F1 to F7). ^c^ significant difference from F11 (F8 to F11). ^d^ significant difference from F15 (F12 to F15) (*p*-value < 0.05).

The cationic surfactant CTAB was incorporated with Cremophor RH40 to fabricate positively charged nanoemulsions, facilitating electrostatic interaction with negatively charged HA. Formulations F1–F7, F1, F2, and F6 exhibited phase separation. An increase in CTAB concentration led to a corresponding increase in droplet size (F3 to F5). F5 exhibited the smallest droplet size, measuring 362.7 ± 62.22 nm (polydispersity index [PDI] value: 0.25 ± 0.01). Droplet size remained unchanged with increasing Cremophor RH40 concentration (F5 and F7) at similar CTAB concentrations (Figure 2a).

The zeta potential value was positive when CTAB was incorporated into the system (Figure 2b). Increasing the CTAB concentration did not result in significant changes in zeta potential (F3 to F5). Nanoemulsions with droplet sizes measuring <500 nm exhibited higher positive surface charges (F4 and F5), whereas those with larger droplet sizes had lower zeta potential values (F3 and F7). At comparable CTAB concentrations, increasing the Cremophor RH40 concentration decreased the zeta potential (F5 and F7).

### 2.3. Effect of Cosurfactant on Droplet Size, Size Distribution, and Zeta Potential

The effect of cosurfactants on nanoemulsion properties was investigated using ethanol and polyethylene glycol 400 (PEG400) at concentrations of 1–10% *w*/*w*. As shown in Figure 2 (F8 to F11), ethanol at 1–2% *w*/*w* significantly reduced the droplet size to approximately 100 nm (PDI value: <0.30; F10 and F11). Nevertheless, concentrations of >2% *w*/*w* were ineffective in further reducing droplet size, as observed in F8 and F9. PEG400 also effectively reduced droplet size, particularly at 1–2% *w*/*w* (F14 and F15). Zeta potential values were positive; however, the two cosurfactants did not exhibit a consistent trend. Reducing the PEG400 concentration increased the zeta potential. In contrast to ethanol, an ethanol concentration of 10% *w*/*w* decreased the zeta potential. However, when reduced to <5% *w*/*w*, the zeta potential did not differ significantly. In further experiments, F11 and F15 were used as representative nanoemulsions for coating with the HA solution.

### 2.4. Effect of Nanoemulsion–Hyaluronic Acid Ratio on Droplet Size, Size Distribution, and Zeta Potential

**Figure 3:**
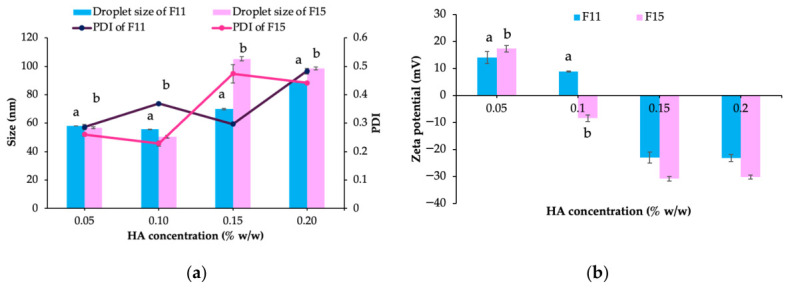
Droplet size, size distribution (polydispersity index [PDI]) (**a**) and zeta potential (**b**) of the nanoemulsions prepared with varying hyaluronic acid (HA) concentrations. ^a^ significant difference within F11 HA-coated formulations. ^b^ significant difference within F15 HA-coated formulations (*p*-value < 0.05).

The various HA concentrations (0.05%, 0.10%, 0.15%, and 0.20% *w*/*w*) were incorporated in F11 and F15. As shown in Figure 3, with increasing HA concentration, the droplet size significantly increased, whereas the zeta potential decreased. The PDI value tended to increase with increasing HA concentration in both F11 and F15 HA-coated formulations.

### 2.5. Molecular Dynamics Simulation Study

**Figure 4:**
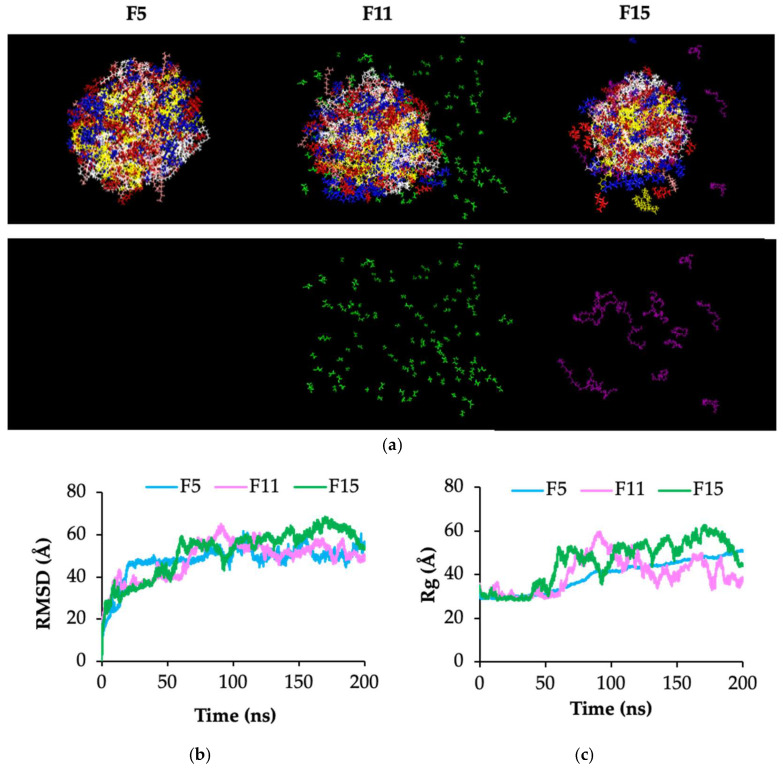
Molecular dynamics simulation of F5, F11, and F15 at 200 ns (pink = cetyltrimethylammonium bromide [CTAB], white = Cremophor RH40, yellow = trilaurin, blue = menthone, red = menthol, green = ethanol, purple = polyethylene glycol 400 [PEG400]) (**a**). Root mean square deviation (RMSD) (**b**) and radius of gyration (Rg) (**c**) of F5, F11, and F15.

To elucidate the molecular organization within the nanoemulsion, MD simulations of F5, F11, and F15 were conducted using AMBER20 for 200 ns. Representative snapshots at 200 ns obtained after removing water molecules are shown in Figure 4a. All aggregations exhibited a distinct spatial organization governed by molecular polarity. The nonpolar component trilaurin was confined to the inner core of the assembly. Amphiphilic components, including Cremophor RH40 and CTAB, were preferentially distributed at the oil–water interface. PEG400 was predominantly localized at the oil–water interface, with a minor fraction distributed in the aqueous phase. Ethanol was distributed in the aqueous phase and the interfacial region, with a higher proportion in the aqueous phase compared to PEG400.

As illustrated in Figure 4b,c, the root mean square deviation (RMSD) and radius of gyration (Rg) of aggregation with and without cosurfactant were compared. RMSD was applied to evaluate conformational stability [15], whereas Rg was used to describe the degree of molecular compactness [16].

Within the first 50 ns, F5 exhibited the highest RMSD and reached a steady state with minimal fluctuations. F11 and F15 showed noticeable RMSD fluctuations during the simulation. The RMSD of F15 increased continuously with time, whereas that of F11 exhibited a more stable trajectory. The Rg values of F11 and F15 showed higher fluctuation than those of F5. F11 exhibited a relatively stable Rg with minimal fluctuation, whereas F15 showed a gradual increase in Rg over time.

### 2.6. Entrapment Efficiency After Glabridin Loading

F11 HA-coated at 0.15% *w*/*w* (F11_HA) was used as the representative nanoemulsion for this analysis because it had the smallest droplet size and narrow size distribution. It was loaded with an excessive amount of GLA. The entrapment efficiency was 91.65% ± 1.52% *w*/*w* (Table 1).

### 2.7. Droplet Size and Zeta Potential After Glabridin Loading

GLA at 0.50% *w*/*w* was loaded onto F11_HA. As shown in Table 1, after incorporating GLA (F11_HA_GLA), the droplet size remained comparably low at 67.63 ± 0.33 nm and PDI increased slightly. The zeta potential increased from −23.00 ± 2.08 mV to −10.83 ± 1.91 mV.

### 2.8. Transmission Electron Microscopy Images

**Figure 5:**
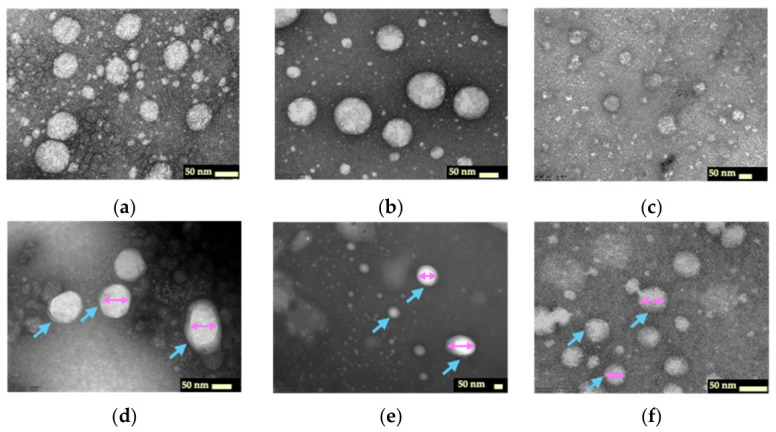
Transmission electron microscopy (TEM) images of nanoemulsions prepared with different hyaluronic acid concentrations: uncoated (F11) (**a**), 0.05% *w*/*w* (**b**), 0.10% *w*/*w* (**c**), 0.15% *w*/*w* (**d**), 0.20% *w*/*w* (**e**), and HA-coated glabridin nanoemulsion (F11_HA_GLA) (**f**). Blue arrows represent the coated hyaluronic acid, and pink arrows indicate the oil core.

The morphology of F11 and its coating with various concentrations of HA was evaluated using transmission electron microscopy (TEM) (Figure 5). The F11 presented an individually spherical shape with a smooth surface (Figure 5a). After being coated with HA (Figure 5b–e), all samples exhibited spherical nanodroplets, with a distinct HA coating clearly observed at 0.15% *w*/*w* concentration, corresponding well with droplet size and zeta potential measurements. After GLA loading, the HA coating was still clearly observed, with a spherical morphology (Figure 5f).

### 2.9. Stability

**Figure 6:**
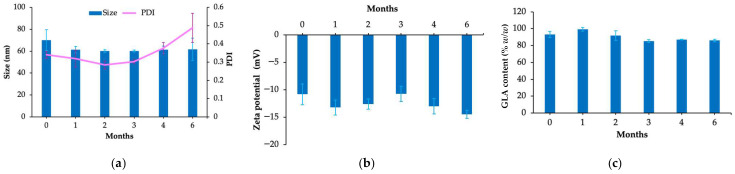
Stability of hyaluronic acid-coated glabridin nanoemulsion (F11_HA_GLA): droplet size, size distribution (polydispersity index [PDI]) (**a**), zeta potential (**b**), and GLA (glabridin) content (**c**).

To assess the stability of F11_HA_GLA, it was stored at 40 °C for 6 months. The droplet size, size distribution, zeta potential, and GLA content were investigated at 1, 2, 3, 4, and 6 months. Droplet size, size distribution, and zeta potential remained stable after storage (Figure 6a,b). The GLA content slightly decreased from 93% to 86% *w*/*w*; however, this reduction remained within the acceptable rate of <10% (Figure 6c).

### 2.10. Cellular Uptake

**Figure 7:**
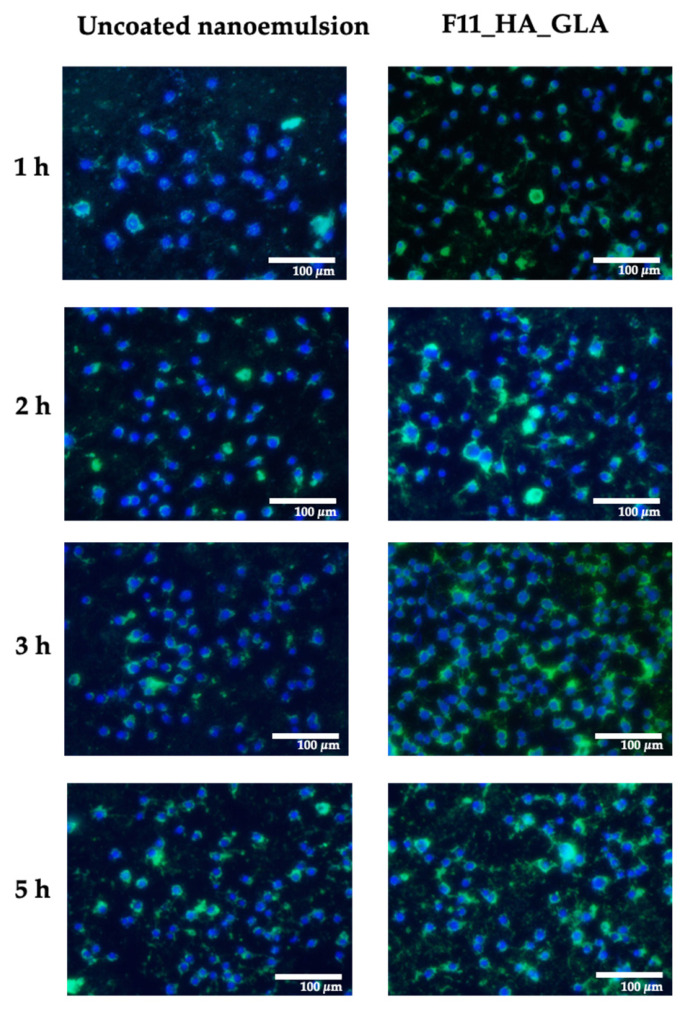
Cellular uptake after treatment with uncoated nanoemulsion and hyaluronic acid-coated glabridin nanoemulsion (F11_HA_GLA) at 1, 2, 3, and 5 h. Green and blue represent the nanoemulsion within macrophage cells and macrophage nuclei, respectively.

Cellular internalization of nanoemulsion droplets may allow direct intracellular delivery of the encapsulated compound, reducing the need for prior release into the surrounding medium. To elucidate the delivery behavior of the nanoemulsion system, cellular uptake was investigated. F11_HA_GLA and the uncoated nanoemulsion (at 0.10% *v*/*v* concentration, below cytotoxic levels) were chosen as representative nanoemulsions for cellular uptake studies (Figure 7). Green fluorescent represents the nanoemulsion within cells whereas blue fluorescent represents the nuclei of the macrophages (RAW 264.7). F11_HA_GLA demonstrated a higher uptake than the uncoated formulation at all time points, as indicated by the greater green fluorescence intensity observed.

### 2.11. Cytotoxicity

**Figure 8:**
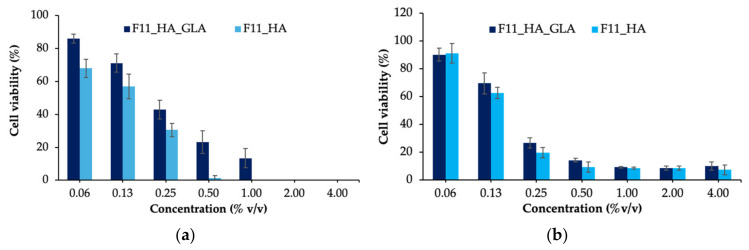
Cell viability of MRC-5 (**a**) and RAW 264.7 (**b**) cells after treatment with hyaluronic acid-coated nanoemulsions (F11_HA) and glabridin loading (F11_HA_GLA).

F11_HA_GLA and base (F11_HA) were selected as representative nanoemulsions for cytotoxicity studies. The cytotoxicity of F11_HA_GLA and F11_HA on RAW 264.7 and normal lung fibroblast (MRC-5) cells was evaluated using the 4,5-dimethylthiazol-2-yl)-2,5-diphenyltetrazolium bromide (MTT) assay (Figure 8). At concentrations <0.13% *v*/*v*, both cell types exhibited high viability (>70%), indicating low cytotoxicity. The base formulation showed a higher cytotoxicity than the nanoemulsion at all tested concentrations for both cell types.

### 2.12. Anti-Inflammatory Activity

**Figure 9:**
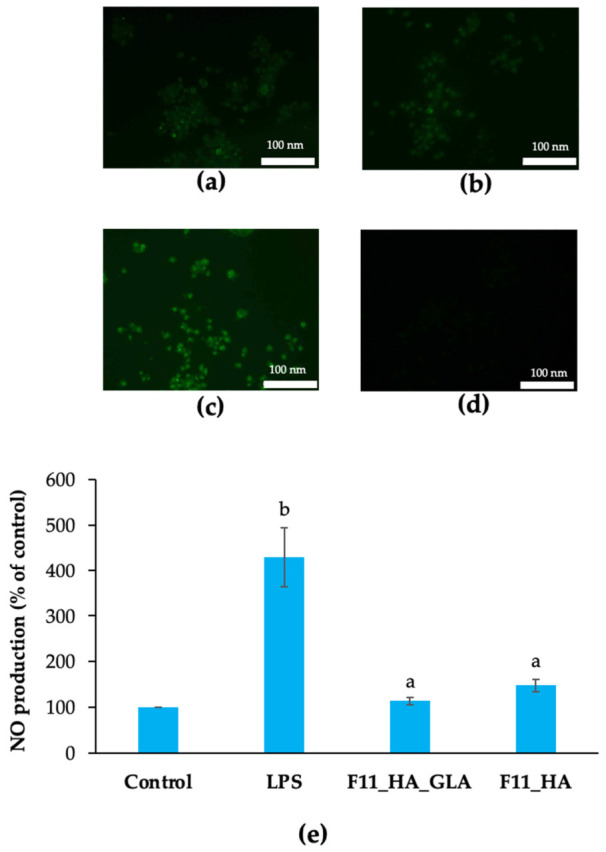
Reactive oxygen species (ROS) production by RAW 264.7 cells after exposure to lipopolysaccharide (LPS). Cells were treated with hyaluronic acid-coated glabridin nanoemulsion (F11_HA_GLA) (**a**), base (F11_HA) (**b**), negative control (without nanoemulsion) (**c**), and untreated cells (without LPS and nanoemulsion) (**d**). Nitric oxide (NO) production by RAW 264.7 cells after treatment with different formulations (**e**). ^a^ significant difference from LPS-treated group. ^b^ significant difference from control (*p*-value < 0.05).

Anti-inflammatory studies were performed using ROS and nitric oxide (NO) production as the marker. F11_HA_GLA and F11_HA was selected for the experiment. Microscopy images revealed intense green fluorescence in LPS-stimulated macrophages, indicating elevated intracellular ROS levels. Treatment with F11_HA_GLA and F11_HA markedly reduced the fluorescence intensity. F11_HA_GLA exhibited slightly lower fluorescence intensity than F11_HA (Figure 9a–d).

As shown in Figure 9e, both nanoemulsions significantly reduced NO production compared with the LPS-treated group. F11_HA_GLA demonstrated a slightly stronger inhibitory effect on NO production compared with F11_HA. The percentage of NO production of F11_HA_GLA and F11_HA was 113.96 ± 7.42% and 148.18 ± 13.79% of control, respectively. The cells remained viable (>70%), confirming that the inhibition of NO production could be attributed to the anti-inflammatory effect rather than cytotoxicity.

## 3. Discussion

Nanoemulsions prepared from different PPO:VCO ratios with Cremophor RH40 via the PIT method exhibited variations in droplet size, indicating that the oil phase composition affects the droplet size. This result correlated with the findings of previous studies, wherein droplet sizes measuring <100 nm was produced from suitable essential oil–fixed oil ratios [13,17]. Furthermore, our previous study revealed that the droplet size of nanoemulsions is influenced by the triglyceride concentration [18]. The near-zero zeta potential values may be attributed to the use of nonionic surfactants, which do not contain charge groups and therefore contribute minimally to the surface charge of the nanoemulsion [19]. A comparable outcome was observed in our earlier work, wherein the use of Cremophor RH40 and Tween led to nanoemulsions exhibiting near-neutral values [20].

The addition of CTAB increased droplet size in a concentration-dependent manner, possibly due to the disruption of the hydration of the polyoxyethylene head group of Cremophor RH40 [21]. This interference may alter the surfactant film curvature, inducing the formation of larger droplets, which contrasts with the results reported by Wang et al. [22]. The study revealed that the addition of the cationic surfactant did not induce any significant change in droplet size in pentaethylene glycol monododecyl ether nanodroplets. However, cationic surfactants may induce an increase in droplet size by modifying the interfacial film, such as by enhancing its rigidity or diminishing its ability to lower interfacial tension [22,23]. These findings indicated that incorporating CTAB into nanoemulsions containing Cremophor RH40, PPO, and VCO promoted a larger droplet size when prepared using the PIT method. Notably, increasing the Cremophor RH40 concentration in the presence of CTAB did not reduce the droplet size, possibly because the Cremophor RH40 concentration exceeded the critical micelle concentration, thus causing surfactant aggregation in the absence of oil and promoting Ostwald ripening [24,25]. Regarding zeta potential, the zeta potential was positive, which is consistent with previous studies. CTAB contributed to a positive zeta potential by accumulating on the oil droplet surface, with the effect increasing at higher concentrations [26]. Nevertheless, in this study, increasing the CTAB concentration did not result in significant changes in zeta potential, possibly due to the use of CTAB concentrations near the critical micelle concentration. At concentrations near or above the critical micelle concentration, the zeta potential tends to plateau or increase only slightly [27,28]. An increase in Cremophor RH40 concentration led to a reduction in zeta potential at comparable CTAB concentrations, which can be attributed to the neutral charge of Cremophor RH40.

The addition of cosurfactants, such as ethanol and PEG400, to the nanoemulsion system lowers interfacial tension and alters the structure of the interfacial film. These effects resulted in smaller droplet sizes and narrower size distribution [29,30]. Nevertheless, an excessive amount of cosurfactant can induce droplet coalescence and compromise the overall stability of the nanoemulsion system [29]. Ethanol is more effective than PEG400 as a cosurfactant in nanoemulsions due to its lower molecular weight and superior ability to reduce interfacial tension. It promotes smaller droplet formation by thinning the interfacial film and enhancing its flexibility. It also alters the solvent properties, thereby promoting a more efficient surfactant arrangement at the interface [31,32]. In contrast, PEG400 is less efficient because of its higher molecular weight and viscosity. When PEG400 or ethanol was used as a cosurfactant to a cationic surfactant in a nanoemulsion system, the zeta potential was primarily influenced by the cationic surfactant. PEG400 and ethanol, which are nonionic, do not contribute significantly to the net surface charge [33].

Although CTAB may interfere with the nanoemulsion formation of Cremophor RH40 via the PIT method, the addition of a suitable ethanol concentration as a cosurfactant enabled phase inversion efficiency. Upon HA incorporation, its anionic nature promotes adsorption onto positively charged oil droplets, resulting in a negatively charged coating on the droplet surface. Consequently, the nanoemulsion exhibited a larger droplet size and a negative zeta potential [34,35]. TEM images confirmed the presence of the HA coating layer. These results are consistent with those reported by Yang et al. [36], who observed that the addition of HA to positively charged nanostructured lipid carriers caused a marked decrease in zeta potential due to the negative charge of HA, along with an increase in droplet size.

MD simulations suggest that the greater distribution of ethanol in the aqueous phase, relative to the PEG400 system, contributes to the smaller droplet size. F5 possessed higher structural stability and tended to aggregate into a single large particle compared with systems containing cosurfactants (F11 and F15). In contrast to F11 and F15, no aggregation into a single large particle was indicated by RMSD behavior during the simulation. The increased fluctuation in Rg for F11 and F15 implies that the aggregates undergo continuous rearrangement, leading to oil separation into a small droplets. F15 exhibited a less compact structure than F11, as indicated by a gradual increase in Rg over time. This may lead to oil phase separation and a broader size distribution, promoting Ostwald ripening and resulting in larger oil droplets compared with F11. The results are consistent with the size analyzer results showing smaller droplet sizes for F11 and F15 compared to F5, with F11 possessing the smallest droplet size. The presence of a cosurfactant likely contributed to droplet size reduction through its incorporation at the oil–water interface, altering the interfacial arrangement of Cremophor RH40, enhancing interfacial flexibility, and altering solvent properties. It was correlated with previous reports revealing that the changes in the surrounding chemical environment can perturb intermolecular interactions and hydration structure. This results in reduced structural compactness and increased conformational flexibility [37].

After GLA loading, the high entrapment efficiency might be the result of GLA solubility. GLA exhibits low solubility in oils, but it is soluble in surfactants [38]. The presence of surfactants, Cremophor RH40 and CTAB, might enhance the solubility of GLA in the oil phase, thereby contributing to the observed high entrapment efficiency. The droplet size remained similar to that before loading with PDI below 0.5, indicating a narrow size distribution [39]. Zeta potential was slightly increased. This increase might be attributed to the presence of GLA, a neutral molecule with no net charge. When incorporated into the nanoemulsion oil droplets, it dilutes or shields the negatively charged surface, thereby reducing the surface charge density and resulting in a relatively lower zeta potential.

In addition to favorable physicochemical properties and high entrapment efficiency, HA-coated GLA nanoemulsion was also showed good physicochemical stability. It may be attributed to the presence of triglycerides, cationic and nonionic surfactants. The presence of VCO may inhibit Ostwald ripening, producing stable droplets that are resistant to this phenomenon. Prior studies have indicated that the incorporation of medium- and long-chain triglycerides can suppress Ostwald ripening [13,40,41]. Furthermore, the incorporation of cationic surfactants and HA in the formulation enhances electrostatic repulsion between droplets, thus preventing coalescence and increasing stability [26,42]. The degradation of GLA is affected by factors such as temperature, light exposure, humidity, pH, and the use of solvents and oxidizing agents. The presence of surfactants around the oil droplets may also protect GLA from environmental factors, while causing only a slight decrease in its content. Previous studies support the ability of nanoemulsions to enhance the stability of active ingredients. The nanoscale oil droplets form a protective barrier, thereby effectively minimizing exposure to environmental stressors, which accelerate the degradation of sensitive compounds [43,44,45].

HA coating enhanced the macrophage uptake of the nanoemulsion. It can facilitate binding to the CD44 receptor, promoting droplet internalization via receptor-mediated endocytosis. This result is in agreement with previous studies. Several studies have found that HA-coated nanocarriers enhanced cellular uptake, especially in CD44-overexpressing cells, such as macrophages. Kamat et al. [46] reported that HA nanoparticles were substantially taken up by activated THP-1 macrophages. Similarly, Wang et al. [47] demonstrated that HA-functionalized nanoparticles preferentially targeted M1 macrophages, with uptake levels 3.7-fold higher than those of normal macrophages.

The cytotoxicity of the base may be attributed to the presence of surfactants, Cremophor RH40 and CTAB, which disrupt cellular membrane integrity, potentially causing membrane destabilization [48]. The lower cytotoxicity of the F11_HA_GLA may be attributed to GLA, which can protect cells from surfactant-induced damage, resulting in higher cell viability following treatment. Several studies have shown that GLA reduced cytotoxicity and prevented cell death under oxidative stress or exposure to toxic agents. For instance, it can protect MC3T3-E1 osteoblastic cells [49] and attenuate cytotoxicity on MC3T3-E1 osteoblastic cells induced by methylglyoxal and antimycin A [50]. The results suggested that HA-coated nanoemulsions containing GLA have low cytotoxicity; however, further clinical investigations are warranted to confirm their actual safety.

The HA-coated GLA nanoemulsion may have exerted anti-inflammatory activity by suppressing the generation of ROS and NO. The activity was a contribution from GLA, while the nanoemulsion system also enhances the overall effect. The concurrent reduction in ROS and NO is mechanistically relevant. NO can react with ROS to generate reactive nitrogen species, which contribute to oxidative and nitrosative stress. Therefore, the simultaneous decrease in both mediators suggests that F11_HA_GLA may reduce inflammation and cellular damage. This result is consistent with previous studies demonstrating that GLA is an effective anti-inflammatory agent by inhibiting the expression of several cytokines and chemokines, as well as reducing NO [51,52] and ROS production [53].

## 4. Materials and Methods

### 4.1. Materials

GLA (Lot No. V241VG) and CTAB (Lot No. OGHB23) were purchased from MySkin Recipe (Bangkok, Thailand). PPO (Lot No. AB1601) was purchased from Krungthep Chemi (Bangkok, Thailand). VCO was purchased from Central Food Retail Company (Bangkok, Thailand). Cremophor RH40 (Lot No. 30696747G0) was purchased from P.C. Drug Center (Bangkok, Thailand). Absolute ethanol (Lot No. K47670583611) was purchased from Merck (Darmstadt, Germany). PEG400 (Lot No. 140353943108P06) was purchased from Fluka Analytical (Buchs, Switzerland). RAW 264.7 cells (Lot No. 70046149) were purchased from the American Type Culture Collection (Manassas, VA, USA). MRC-5 was kindly provided by the Faculty of Pharmacy, Silpakorn University (Nakhon Pathom, Thailand). All other chemicals were reagent-grade and used as received.

### 4.2. Nanoemulsion Preparation

The GLA nanoemulsion was prepared using the PIT method according to the procedure described in our previous reports [40,54]. VCO, GLA, and Cremophor RH40 were mixed and heated to 60 °C. PPO was incorporated into the oil phase as the last component and exposed to 60 °C for the minimum possible time. The aqueous phase, consisting of PEG400 or ethanol, CTAB, and water, was heated to 60 °C and mixed thoroughly. Ethanol was introduced as the final component in the aqueous phase with minimal exposure to 60 °C. Both phases were homogenized using an IKA T25 digital homogenizer (IKA, Staufen, Germany) at 3800 rpm for 5 min. Thereafter, the resulting nanoemulsion was mixed with HA solution (0.05%, 0.10%, 0.15%, and 0.20% *w*/*w*) at a 1:10 ratio. The HA-coated nanoemulsion was subsequently evaluated. The nanoemulsion formulations are shown in Table 2.

### 4.3. Determination of Droplet Size, Size Distribution, and Zeta Potential

Dynamic light scattering (Malvern Instruments, Worcestershire, UK) was performed to determine the droplet size, size distribution, and zeta potential of the nanoemulsions. Data were expressed as mean ± standard deviation (SD) (*n* = 3).

### 4.4. Molecular Dynamics Simulation Study

Atomic coordinates of menthol and menthone (PPO constituents), CTAB, ethanol, and PEG400 were sourced from the Protein Data Bank (identifiers: 8UXY, 8AEJ, 1KPG, 8ANG, and 7Y1X, respectively). The molecular structures of trilaurin (main composition in VCO) and Cremophor RH40 were generated manually using GaussView 06 (Gaussian, Wallingford, CT, USA) and geometry-optimized at the B3LYP/6-311G* level. Parameterization of all components, including the generation of topology and force field modification (frcmod) files, was performed using the antechamber and parmchk2 modules implemented in AMBER20 [55]. Three systems were assembled with defined mole ratios using the LEaP module: F5, menthol–menthone–Cremophor RH40–CTAB–trilaurin–water (23:16:4:3:6:4389); F11, menthol–menthone–Cremophor RH40–ethanol–CTAB–trilaurin–water (23:16:4:22:3:6:4333); and F15, menthol–menthone–Cremophor RH40–PEG400–CTAB–trilaurin–water (23:16:4:3:3:6:4333). A particle mesh Ewald method was employed to treat nonbonded and long-range electrostatic interactions, using a cutoff distance of 12 Å. The system was gradually heated from 0 to 333 K over 100 ps in the NVT ensemble, followed by equilibration in the NPT ensemble at 333 K for 400 ps. Subsequently, production simulations were conducted for 200 ns under NPT conditions at 333 K. Trajectory analyses, including RMSD and Rg, were carried out using cpptraj, while molecular visualization was performed with visual MD [56].

### 4.5. Determination of Entrapment Efficiency and Glabridin Content

High-performance liquid chromatography (HPLC; Shimadzu, Kyoto, Japan) was performed to determine the ability of a carrier to encapsulate drug (entrapment efficiency). An excessive amount of GLA (2% *w*/*w*) was incorporated into the oil phase and blended with the aqueous phase, as described in Section 4.2. Subsequently, 1 g of the nanoemulsion was accurately weighed and diluted with deionized water to a final volume of 50 mL in a volumetric flask. Then, 5 mL of the resulting dispersion was withdrawn and filtered through a 0.45-µm nylon membrane filter, and the unentrapped GLA retained on the membrane was dissolved in methanol and filtered through a 0.45-µm nylon membrane filter. Concentration analysis was performed using a 250 mm × 4.6 mm C18 column (SGE Analytical Science, Melbourne, Australia) with an isocratic mobile phase of water and acetonitrile (30:70, *v*/*v*) at 30 °C. Detection was performed using an ultraviolet detector at 280 nm, with a flow rate of 1 mL/min. The calibration curve ranged from 125 to 750 µg/mL based on the peak area. Entrapment efficiency was calculated using the following Equation (1) (*n* = 3):

(1) $$Entrapment\;efficiency\left(\%w/w\right)=100\times \frac{GLA\;total-GLA\;filtered\;residue}{GLA\;total}$$

To determine the GLA content of the nanoemulsion, which represents the actual amount of GLA present in the final formulation, an accurately weighed amount of nanoemulsion was diluted with isopropyl alcohol and analyzed by HPLC using the previously described conditions. The GLA content was calculated using Equation (2):

(2) $$GLA\;content\left(\%w/w\right)=100\times \frac{Amount\;of\;GLA\;in\;formulation}{Theoretical\;GLA}$$

### 4.6. Transmission Electron Microscopy Image Analysis

Representative nanoemulsions were placed on a copper grid and negatively stained with 2% uranyl acetate. Their morphology was subsequently examined using an 80–100 kV TecnaiG2 20 TEM (Philips, Amsterdam, The Netherlands).

### 4.7. Stability Test

The stability of the optimized HA-coated GLA nanoemulsions was assessed by storing samples (*n* = 3) in glass vials at 40 ± 2 °C for 6 months. After storage, the nanoemulsions were evaluated for droplet size, size distribution, zeta potential, and GLA content using the methods described in Section 4.3 and Section 4.5.

### 4.8. Cellular Uptake Assessment

Cellular uptake was investigated using RAW 264.7 cells. The green fluorescent probe Coumarin 6 was incorporated into the oil phase of the nanoemulsion. The cells were seeded into 6-well plates at a density of 5 × 10^5^ cells/well and incubated at 37 °C under 5% CO_2_ for 24 h. LPS (1 µg/mL) was induced into the plate for 2 h at 37 °C. The cells were separately treated with 0.1% *v*/*v* of GLA nanoemulsion or HA-coated GLA nanoemulsion. After 1, 2, 3, and 5 h, the treated media were removed, and the cells were washed thrice with 1 mL of phosphate-buffered saline (PBS). Treated cells were permeabilized with 0.2% *v*/*v* Triton-X-100, fixed in 4% *v*/*v* paraformaldehyde for 10 min, and stained with 300 nM Hoechst 33258 for 10 min in a dark environment. The stained cells were subsequently washed with PBS and examined using an inverted fluorescent microscope (Nikon, Tokyo, Japan) equipped with blue and green filters.

### 4.9. Cytotoxicity Study

The cytotoxicity test was performed on MRC-5 and RAW 264.7 cell lines using the MTT assay. Cells were seeded at a density of 1 × 10^4^ cells/well in 96-well plates and incubated for 24 h. Subsequently, the cells were treated with the base and HA-coated glabridin nanoemulsion at concentrations of 0.06–4.00% *v*/*v*. After 24 h, 50 µL of 50 mg/mL MTT solution was added to each well after removal of the treatment media. The plates were covered with aluminum foil to protect them from light and incubated for 3 h at 37 °C under 5% CO_2_. Subsequently, 100 µL dimethyl sulfoxide was replaced to each well to solubilize the purple formazan crystals. Absorbance was measured at 570 nm wavelength using a microplate reader (BMG Labtech, Ortenberg, Germany) to assess cell viability. The percentage of viable cells was calculated using Equation (3) (*n* = 3):

(3) $$Cell\;viability(\%)=100\times \frac{Mean\;absorbance\;of\;treated\;cell}{Mean\;absorbance\;of\;untreated\;cell}$$

### 4.10. Analysis of Reactive Oxygen Species Production

The anti-inflammatory effects were evaluated by observing intracellular ROS levels using a 2′,7′-dichlorodihydrofluorescein diacetate probe. A 6-well plate containing RAW 264.7 cells at 1 × 10^5^ cells/well was incubated for 24 h. Subsequently, the HA-coated GLA nanoemulsion or base (0.1% *v*/*v*) was added to the plates, followed by LPS (1 μg/mL). LPS was used in the negative control group. Thereafter, cells were treated with 10 μM 2′,7′-dichlorodihydrofluorescein diacetate for 0.5 h at 37 °C in the dark. Following incubation, the plate was rinsed twice with PBS and observed under a fluorescence microscope (Nikon, Tokyo, Japan).

### 4.11. Determination of Nitric Oxide Production

RAW 264.7 cells were seeded in 96-well plates (5 × 10^4^–6 × 10^4^ cells/well) and incubated for 24 h. The cells were then treated with the HA-coated GLA nanoemulsion or base (0.1% *v*/*v*) and stimulated with LPS (1 μg/mL) for 24 h. After incubation, the culture supernatants were collected, and NO production was determined by measuring nitrite levels using the Griess reagent. Absorbance was measured at 540 nm using a microplate reader (BMG Labtech, Ortenberg, Germany), and nitrite concentration was calculated using a sodium nitrite standard curve. NO production was expressed as a percentage of the control (untreated cells without LPS and nanoemulsions) and presented as mean ± SD (*n* = 3).

### 4.12. Statistical Analysis

Statistical analyses were performed using SPSS version 10.0 for Windows (SPSS Inc., Chicago, IL, USA). Data comparisons were conducted using one-way analysis of variance (ANOVA), followed by Tukey’s post hoc test, with significance evaluated at a 95% confidence level.

## 5. Conclusions

In this study, an HA-coated nanoemulsion containing GLA was successfully developed using the PIT method. The optimized cationic nanoemulsion consisted of PPO:VCO at a 60:40 ratio (10% *w*/*w*), Cremophor RH40 (10% *w*/*w*), CTAB (1% *w*/*w*), and ethanol (1% *w*/*w*) as key components. Ethanol demonstrated superior performance as a cosurfactant compared with PEG400. MD simulations confirmed the contribution of ethanol as a cosurfactant to the formation of smaller nanoemulsions. The optimal HA concentration was 0.15% *w*/*w*, with a nanoemulsion–HA ratio of 1:10. The final formulation exhibited a small droplet size, a narrow size distribution and a negatively charged zeta potential. It also enhanced macrophage uptake, showed low cytotoxicity, and effectively inhibited ROS and NO production. These findings highlight the potential of the PIT method, in combination with Cremophor RH40, CTAB, and ethanol, to produce the HA-coated, glabridin nanoemulsion as a promising delivery platform for enhanced macrophage uptake and inflammation alleviation.

## Figures and Tables

**Table 1:** Properties of HA-coated nanoemulsions before (F11_HA) and after (F11_HA_GLA) glabridin loading.

Sample	Entrapment Efficiency (% *w*/*w*)	Droplet Size (nm)	Zeta Potential (mV)	PDI
F11_HA	-	69.98 ± 0.48	−23.00 ± 2.08	0.30 ± 0.00
F11_HA_GLA	91.65 ± 1.52	67.63 ± 0.33	−10.83 ± 1.91	0.36 ± 0.01

**Table 2:** Formulations of nanoemulsions.

Composition (% *w*/*w*)	F1	F2	F3	F4	F5	F6	F7	F8	F9	F10	F11	F12	F13	F14	F15
PPO	6.0	6.0	6.0	6.0	6.0	6.0	6.0	6.0	6.0	6.0	6.0	6.0	6.0	6.0	6.0
VCO	4.0	4.0	4.0	4.0	4.0	4.0	4.0	4.0	4.0	4.0	4.0	4.0	4.0	4.0	4.0
Cremophor RH40	10.0	10.0	10.0	10.0	10.0	5.0	15.0	10.0	10.0	10.0	10.0	10.0	10.0	10.0	10.0
CTAB	15.0	10.0	5.0	2.0	1.0	1.0	1.0	1.0	1.0	1.0	1.0	1.0	1.0	1.0	1.0
Ethanol	-	-	-	-	-	-	-	10.0	5.0	2.0	1.0	-	-	-	-
PEG400	-	-	-	-	-	-	-	-	-	-	-	10.0	5.0	2.0	1.0
Water	65	70	75	78	79	84	74	69	74	77	78	69	74	77	78

## Data Availability

The original contributions presented in this study are included in the article. Further inquiries can be directed to the corresponding author.
